# Bioinspired enamel repair via inorganic ionic polymerization using calcium phosphate ionic clusters and nano-hydroxyapatite

**DOI:** 10.1038/s41598-025-06434-7

**Published:** 2025-06-20

**Authors:** Marwa A. Elkhouly, Mahmoud M. Emara, Norhan Nady, Ahmed S. Elkadi, Sherif H. Kandil

**Affiliations:** 1 Materials Science Department, Institute of Graduate Studies and Research, Alexandria University, El Shatby, Alexandria 21526 Egypt; 2 Chemistry Department, Faculty of Science, Alexandria University, Baghdad Street, P.O. Box 21511, Moharam Bey, Alexandria Egypt; 3 Polymeric Materials Research Department, Advanced Technology and New Materials Research Institute (ATNMRI), City of Scientific Research and Technological Applications (SRTA-City), New Borg El-Arab City, Alexandria 21934 Egypt; 4 Conservative Dentistry Department, Faculty of Dentistry, Alexandria University, Azarita, Alexandria 21521 Egypt

**Keywords:** Enamel repair, Hydroxyapatite, Calcium phosphate ionic clusters, Inorganic ionic polymerization, Bioinspired materials, Dental biomaterials

## Abstract

Traditional restorative materials often fail to integrate seamlessly with natural tooth structures due to differences in chemical composition, leading to microleakage and related clinical problems. This study aims to create a material entirely composed of calcium phosphates, the main component of dental enamel, to repair minor enamel cavities. Inspired by the biomineralization process and employing the inorganic ionic polymerization strategy, a cohesive calcium phosphate mass was developed to repair minor enamel cavities. Calcium phosphate ionic clusters (CPICs) and three types of calcium phosphate powder were utilized; (bone-derived and synthetic hydroxyapatite; (BHA and SHA), and dried CPICs. Two different techniques, namely layer by layer (LbL) and premixing (PM), were employed to mix CPICs with one type of calcium phosphate powder to form a cohesive mass. Different analysis techniques were used including FTIR, XRD, SEM and TEM. Among the tested approaches, the mass formed by mixing BHA with CPICs using the PM technique demonstrated superior integration with enamel walls and infiltration of calcium phosphate particles into enamel. To the best of our knowledge, repairing cavitated enamel defects using a bioinspired approach with a material composed entirely of calcium phosphate has not yet been achieved.

## Introduction

Dental enamel is a highly mineralized dental tissue that consists of almost 96 wt% calcium phosphates in the form of hydroxyapatite (HAP) crystals^1^. In the oral cavity, destruction of the enamel surface occurs due to different factors and may lead to enamel cavitation^2^. Traditionally, restorative materials like amalgam, composite resins, or ceramics are used to restore these cavitated lesions. The chemical and physical structure of these materials differ from those of natural enamel. These compositional and structural differences lead to integration failure between the restoration and tooth structure^3^. Therefore, novel repair materials with tightly bonded interfaces and minimal microleakage are required. Recently, materials for hard tissue repair have been designed and developed by drawing inspiration from the mechanisms of biomineralization^4^. Inorganic ionic polymerization is regarded as a bioinspired methodology for material preparation^5^. This method assembles inorganic ions similarly to polymerization in polymer chemistry. During the polymerization process, capping molecules can prevent polymerization and stabilize the polymer structure^6^. Once these molecules are removed, polymerization can resume.

Capping molecules, are small molecules introduced during chemical synthesis to regulate the growth and stability of forming compounds, particularly nanoparticles or polymers^5^. In restorative materials—particularly those mimicking or integrating with hard tissues like enamel or dentin—capping agents play a key role by temporarily binding to the reactive surfaces of monomers or ionic clusters, thereby inhibiting uncontrolled aggregation. This stabilization enables the retention of desirable nanostructures, which are increasingly utilized in advanced biomimetic applications^7,8^. By adopting this capping technique to stabilize nucleating precursors in bioinspired mineralization, a novel class of bioinspired mineralized materials could be produced^9^. Shao et al., employed this strategy to regenerate enamel by applying calcium phosphate ionic clusters (CPICs) on demineralized enamel surfaces. Calcium phosphate ionic clusters, aggregates measuring a few nanometers in size, served as the precursor solution, with triethylamine (TEA) acting as the capping material or stabilizer. However, the drawback of this technique was the limited thickness of the regenerated enamel layer^8^.

The resemblance between the nano-Hydroxyapatite (nHAP) and the enamel HAP makes it a promising material to be used in dental enamel repair^10^. Hydroxyapatite can be synthesized using different methods, resulting in a variety of crystallite morphologies and sizes depending on the synthesis conditions^11,12^. In contrast to synthetic HAP (SHA), natural HAP derived from transforming biological waste, such as bone-derived HAP (BHA), offers an economical and environmentally friendly alternative^13^. Additionally, natural HAP contains trace elements like carbonate, magnesium, sodium, and zinc, which mirror the composition of HAP which is found in human enamel^14,15^. Other calcium phosphate particles have been utilized to enhance tooth remineralization, including amorphous calcium phosphate (ACP) and β-tricalcium phosphate (β-TCP). Nano ACP particles can facilitate the tooth remineralization process by adjusting the pH of the oral cavity and neutralizing the acidic environment created by cariogenic bacteria^16,17^. β-tricalcium phosphate, one of the crystalline forms of calcium phosphate, displayed significant remineralization potential when topically applied to demineralized enamel^18,19^. As mentioned above, previous studies have successfully repaired demineralized enamel surfaces through remineralization or regeneration using various forms of calcium phosphates, such as ACP, β-TCP, HAP, and CPICs. However, to the best of our knowledge, repairing cavitated enamel defects (~ 500 µm in depth) using a bioinspired approach with a material composed entirely of calcium phosphate has not yet been achieved.

The primary objective of this research is to utilize the inorganic ionic polymerization approach to develop a material composed entirely of calcium phosphates for the repair of minor cavitated enamel defects. Calcium phosphate ionic clusters (CPICs) and three types of calcium phosphate powder were utilized; (bone-derived and synthetic hydroxyapatite; (BHA and SHA), and dried CPICs. Two different techniques, namely layer by layer (LbL) and premixing (PM), were employed to mix CPICs with one type of calcium phosphate powder to form a cohesive mass. Different analysis techniques were used.

## Materials and methods

### Materials

To prepare CPICs, the following chemicals were used; calcium chloride dihydrate (CaCl_2_.2H_2_O; 99.5%), TEA [(C_2_H_5_)_3_N; 99.5%], ethanol (C_2_H_5_-OH; 99.8%), and orthophosphoric acid (H_3_PO_4_; 85%). For SHA preparation, calcium nitrate tetrahydrate [Ca(NO_3_)_2._4H_2_O; 99%], diammonium hydrogen phosphate [(NH_4_)_2_HPO_4_], citric acid anhydrous (C_6_H_8_O_7_; 99.5%) and ammonia water (NH_4_OH; 32%) were used. Modified simulated oral fluid (m-SOF) was produced with sodium fluoride (NaF; 98%), potassium chloride (KCl; 99%), HEPES buffer (C_8_H_18_N_2_O_4_S; 99%, (4-(2-hydroxyethyl)-1-piperazineethane sulfonic acid), which is a zwitterionic sulfonic acid buffering agent, di-potassium hydrogen phosphate anhydrous (K_2_HPO_4_; 99%), and sodium azide (Na_3_N; 99%). All the chemicals were obtained from Alpha Chemika, India and were used without any further purification.

### Specimens’ preparation

The protocol of this in vitro study received approval (Serial Number: 0202041) by the Ethics Committee, Faculty of Medicine, Alexandria University (IRB NO: 00012098, FWA NO: 00018699). All procedures occurred in accordance with relevant ethical guidelines and regulations of Helsinki Declaration. For the collection of extracted teeth, informed consent was obtained from all participants.

Extracted human teeth, obtained for various therapeutic reasons, were collected from the Oral and Maxillofacial Surgery Department at the Faculty of Dentistry, Alexandria University. Teeth were collected from donors aged between 18 and 30 years. Maxillary premolars were selected for inclusion in the study due to their sufficient enamel thickness, which allows for the preparation of suitable enamel slabs. The teeth were cleaned, and sterilized using autoclave sterilization (Autoclave HS33, Getting, Sweden)^20^. Through visual inspection, any carious or defective enamel surfaces were excluded. Only sound enamel surfaces were selected and sectioned to produce enamel slabs. Slabs measuring approximately 2 × 3 mm were obtained from the middle third of the proximal surface to ensure a less curved geometry and to avoid the cervical third, where the enamel is typically thinner.

**Fig. 1:**
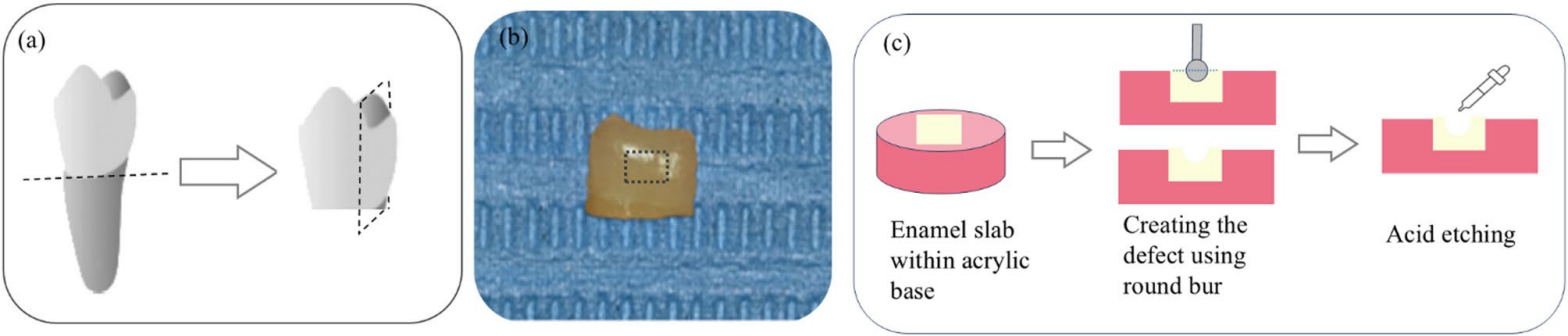
Schematic diagram illustrates enamel slab preparation: (**a**) Separation of the proximal surface; (**b**) Separated proximal surface with a dotted line indicating the area from which the slab was taken; (**c**) Placement of the slab into an acrylic base to facilitate manipulation, and creation of an enamel defect using a 1 mm diameter round bur, with half of the bur’s sphere inserted to achieve a cavity depth of 0.5 mm, followed by acid etching of the cavity.

To facilitate the manipulation of the specimens, each enamel slab was molded inside an acrylic resin mold. All of the specimens were then stored inside 0.2 wt% thymol solution at 4 °C till use^8^. To simulate minor cavitated lesions, a small enamel defect (depth ≃500 µm) was created using a round diamond bur with 1 mm diameter (BR-45, Mani Inc., Tochigi, Japan) mounted on a high-speed handpiece. The depth of the defect was controlled by inserting half of the bur’s spherical head (0.5 mm) into the enamel. Although this method does not allow for precise control of the 500 µm depth due to manual handling, it ensures that the defect remains minimal and confined within the enamel layer—an essential consideration in this study. Acid etching with 37% phosphoric acid gel (H_3_PO_4_; 37%, META BIOMED, Korea) for 30 s, followed by washing and drying, was conducted to clean the enamel surface and remove the smear layer^21^. The preparation of enamel slabs is illustrated in Fig. 1.

### Preparations of bone derivatization

#### Preparation of calcium phosphate ionic clusters (CPICs)

**Fig. 2:**
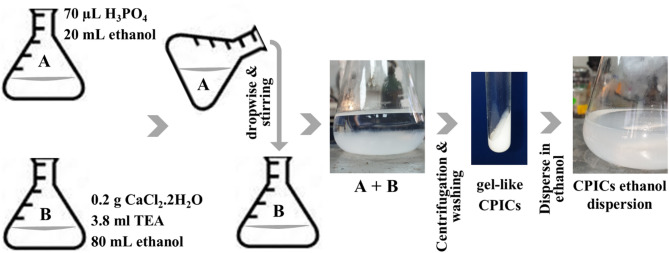
Schematic diagram illustrates the preparation of Calcium phosphate ionic clusters (CPICs).

Calcium phosphate ionic clusters were prepared following the protocol described by Shao et al., as illustrated in Fig. 2^8^. In one flask 0.2 g CaCl_2_·2H_2_O and 3.8 mL TEA were added to 80 mL ethanol, and then that mixture was ultrasonicated for 5 min. In another flask, 70 µL H_3_PO_4_ was added to 20 mL ethanol and stirred thoroughly with a magnetic stirrer. The first mixture was added to the second one drop by drop while stirring. Using centrifugation, gel-like CPICs was collected and then washed twice with ethanol. Finally, 100 mL of ethanol was added to the gel-like CPICs to form a CPICs ethanol solution with a 2 mg/mL concentration.

#### Preparation of dried CPICs powder

According to Shao et al., air drying of the CPICs ethanol solution will produce ACP^8^. An amount of CPICs (2 mg/mL) was dropped onto a glass slab and air-dried at room temperature. The drying process resulted in a white powder that was collected for further investigation and usage.

#### Preparation of synthetic hydroxyapatite (SHA)

**Fig. 3:**
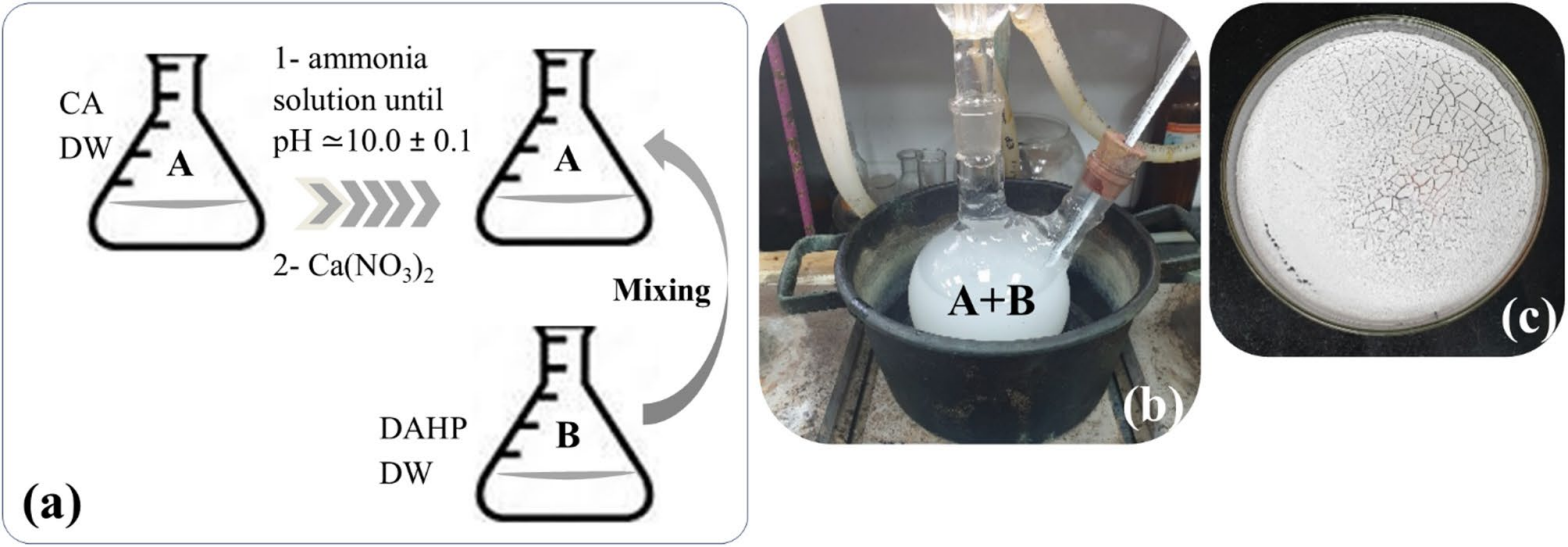
Schematic diagram illustrates the preparation of synthetic hydroxyapatite (SHA). (**a**) Mixing of the chemical components; (**b**) The resulting solution was placed in a water bath at 70 °C and stirred for 24 h; (**c**) The resulting powder obtained after drying the suspension in a thermostatic drying oven at 60 °C for 4 h.

Synthetic hydroxyapatite (SHA) was synthesized following the method described by Snihirova et al., as illustrated in Fig. 3^22^. An amount of 52.54 g citric acid (CA) was added to 250 mL distilled water (DW) and stirred using a magnetic stirrer. By using ammonia water, the solution pH was adjusted to 10.0 ± 0.1, then, an amount of 4.72 g calcium nitrate was added gradually. In another jar, an amount of 2.64 g diammonium hydrogen phosphate (DAHP) was added to 100 mL of DW to form a solution. Next, the two previously mentioned solutions were mixed and stirred together with a magnetic stirrer. Later, the resulting solution was left in a water bath (70 °C) and stirred for 24 h. This procedure resulted in a suspension that was filtered, washed extensively with ethanol and DW, and then kept inside a thermostatic drying oven to dry for 4 h at 60 °C.

#### Preparation of bone-derived hydroxyapatite (BHA)

Hydroxyapatite particles from a natural source (bovine bone) were prepared according to Barakat *et* al.^23^. The bovine bones were collected from the slaughterhouse. Visible tissues on the bone surface were cleaned with water and then ethanol. Next, the bones were dried and then grounded. The resulting powder was then annealed at 750 °C for 6 h in an electrical furnace (Nabertherm 30–3000, Lilienthal, Germany).

#### Preparation of modified simulated oral fluid (m-SOF)

The modified simulated oral fluid (m-SOF) was prepared according to Shao et al.^8^. First, sodium fluoride solution was prepared by adding 0.015 g NaF to 100 mL of distilled water. Next, 0.969 g KCl, 0.47 g HEPES buffer, 0.022 g CaCl_2_.2H_2_O, 0.015 g K_2_HPO_4_ and, 0.0065 g Na_3_N were added to 90 ml distilled water. Finally, 10 ml of the previously prepared NaF solution were added resulting in 100 mL m-SOF.

### Study design

**Fig. 4:**
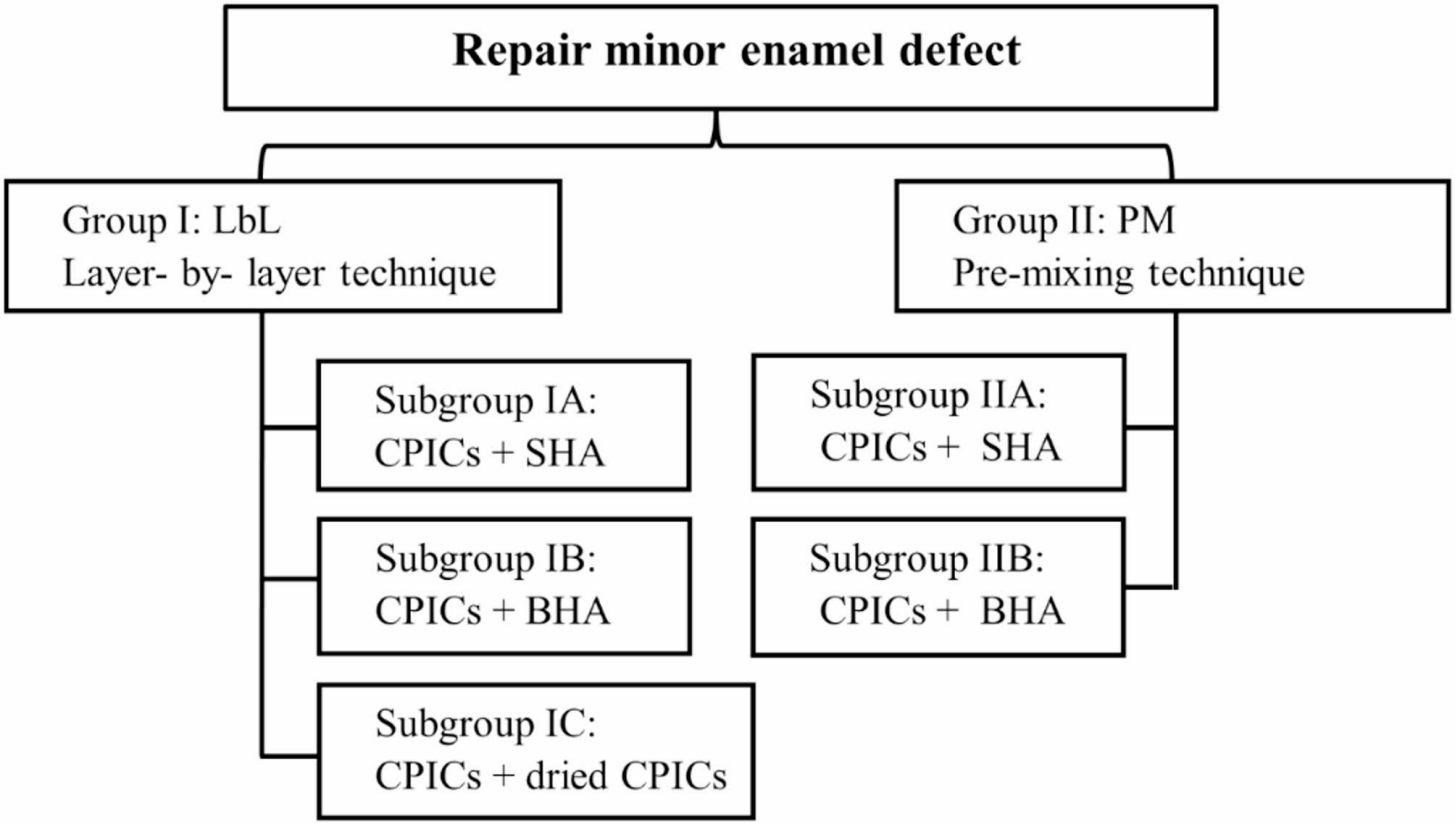
Schematic flowchart representing the study groups and subgroups; Calcium phosphate ionic clusters (CPICs), synthetic hydroxyapatite (SHA), and bone-derived hydroxyapatite (BHA).

To repair minor enamel defects, a cohesive mass was created by mixing an ethanol solution of CIPCs with calcium phosphate particles from various sources. This study used different types of calcium phosphate particles, including BHA, SHA, and dried CPICs. The enamel specimens were divided into two main groups based on the filling technique used, and each group was further subdivided based on the type of calcium phosphate powder utilized. In total, 10 enamel slabs were organized into five subgroups of two slabs each. Each subgroup was treated following the specified protocol and examined using scanning electron microscopy (SEM). Figure 4 illustrates the different study groups and subgroups.

#### Group I. Layer by layer (LbL) technique

A drop of CPICs ethanol solution was placed inside the etched enamel defect. Subsequently, a small quantity of powder was added and compacted with the solution within the defect. This sequence was then repeated, with the addition of another drop of CPICs ethanol solution, until the entire enamel defect was adequately filled. This group was further subdivided into three subgroups based on the type of calcium phosphate powder used, SHA, BHA, or dried C.

#### Group II. Premixing (PM) technique

In this technique, the CPICs ethanol solution and the powder were pre-mixed in a specific ratio for each type of powder to form a paste (0.4 mL CPICs + 0.03 g SHA, 0.4 mL CPICs + 0.9 g BHA). The enamel defect was initially wetted with a drop of CPICs ethanol solution and then filled with the preformed paste as a bulk, differing from the LbL approach used in Group I. This group was subdivided into only two subgroups according to the powder used: SHA and BHA.

In both groups, after filling the enamel defects, the specimens were stored in m-SOF to simulate the oral environment for 48 h and then were examined.

### Instrumental characterization

#### Micro-Fourier transform infrared spectroscopy (FTIR) analysis

To confirm the nature of the three prepared powder types used in this study, functional group identification of the BHA, SHA, and dried CPICs was determined using the FTIR analyzer (Micro-Fourier Transform Infrared Spectroscopy Shimadzu Ft/IR-8400 Spectrophotometer, Japan). The infra-red absorption spectra were measured in the frequency range 4000–400 cm^−1^. To evaluate the structural changes in etched enamel following various treatments, FTIR spectroscopy was performed using a Platinum ATR (Bruker Alpha II, Bruker Optik GmbH, Germany). Spectra were collected in the range of 1500–400 cm^−^1 for untreated etched enamel, enamel treated with CPICs solution, CPICs mixed with BHA, and CPICs mixed with dried CPICs powder.

#### X-ray diffraction (XRD) analysis

X-ray diffraction analysis was performed using D8 Discover XRD (Bruker Co., Germany) to ascertain which phase of the calcium phosphate compound is created during the drying of the CPICs. The X-ray tube was operated at a voltage of 40 kV and a current of 40 mA. The XRD measurements were conducted at a wavelength of 1.54 A°.

#### Scanning electron microscopy (SEM) imaging

The particle size and morphological characterization of the SHA, BHA, and dried CPICs powder were investigated using SEM (JSM-IT 200, JEOL, Tokyo, Japan). In addition, the degree of integration between the enamel surface and the newly formed calcium phosphate material, the size of microleakage, and the amount of calcium phosphate particles infiltrated inside the underlying enamel were examined.

#### Transmission electron microscope (TEM) imaging

Transmission Electron Microscope Imaging using (JEOL–JSM–1400 PLUS, Tokyo, Japan) was conducted to assess the size and particle morphology of the dried CPICs powder. Prior to TEM examination, the powders were dispersed in ethanol and subjected to 10 min of ultrasonication to form a nano-dispersion solution. Subsequently, the solution was loaded onto a copper grid, stained with 2% uranyl acetate to enhance the contrast, and then it was allowed to dry at room temperature.

## Results and discussion

Despite advancements in restorative dentistry, the chemical composition and crystal structure of the current restorative materials are significantly different from the natural biominerals found in tooth enamel. This discrepancy prevents a completely sealed interface with the remaining enamel surface, often leading to post-repair problems. Therefore, there is an urgent need for materials that closely resemble enamel to achieve optimal treatment results^3^.

This study aimed to repair minor enamel defects using a material that replicates the chemical structure of natural dental enamel to achieve superior sealing and promote remineralization. To accomplish this, calcium phosphate particles from various sources (BHA, SHA, and dried CPICs) were prepared and mixed with an ethanol solution of TEA-stabilized CPICs to create a cohesive mass. This mass was then applied to the enamel defects using either the LbL or PM techniques.

### Characterization of the calcium phosphate powders

#### The bone-derived hydroxyapatite (BHA)

**Fig. 5:**
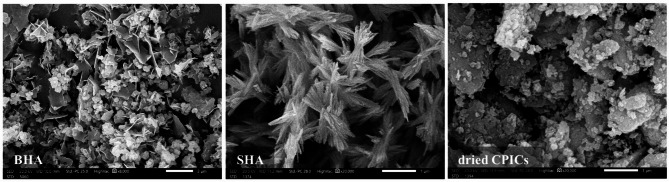
SEM images of bone-derived hydroxyapatite (BHA) (scale bar 2 µm, 8000X), synthetic hydroxyapatite (SHA) (scale bar 1 µm, 20,000X), and dried calcium phosphate ionic clusters (CPICs) (scale bar 1 µm, 20,000X).

The SEM image of BHA (Fig. 5) reveals distinct particle morphologies, including both globular and flake structures. The globular particles exhibit diameters ranging from 100 to 300 nm, while the flakes exhibit a thin thickness of less than 100 nm. According to ISO, I. (2015), TS 80004-1, nano powders are defined as powders with one or more external dimensions in the nanoscale, typically between 1 and 100 nm^24^. Therefore, based on these findings, it can be concluded that BHA is composed of both micro- and nano-particles.

**Fig. 6:**
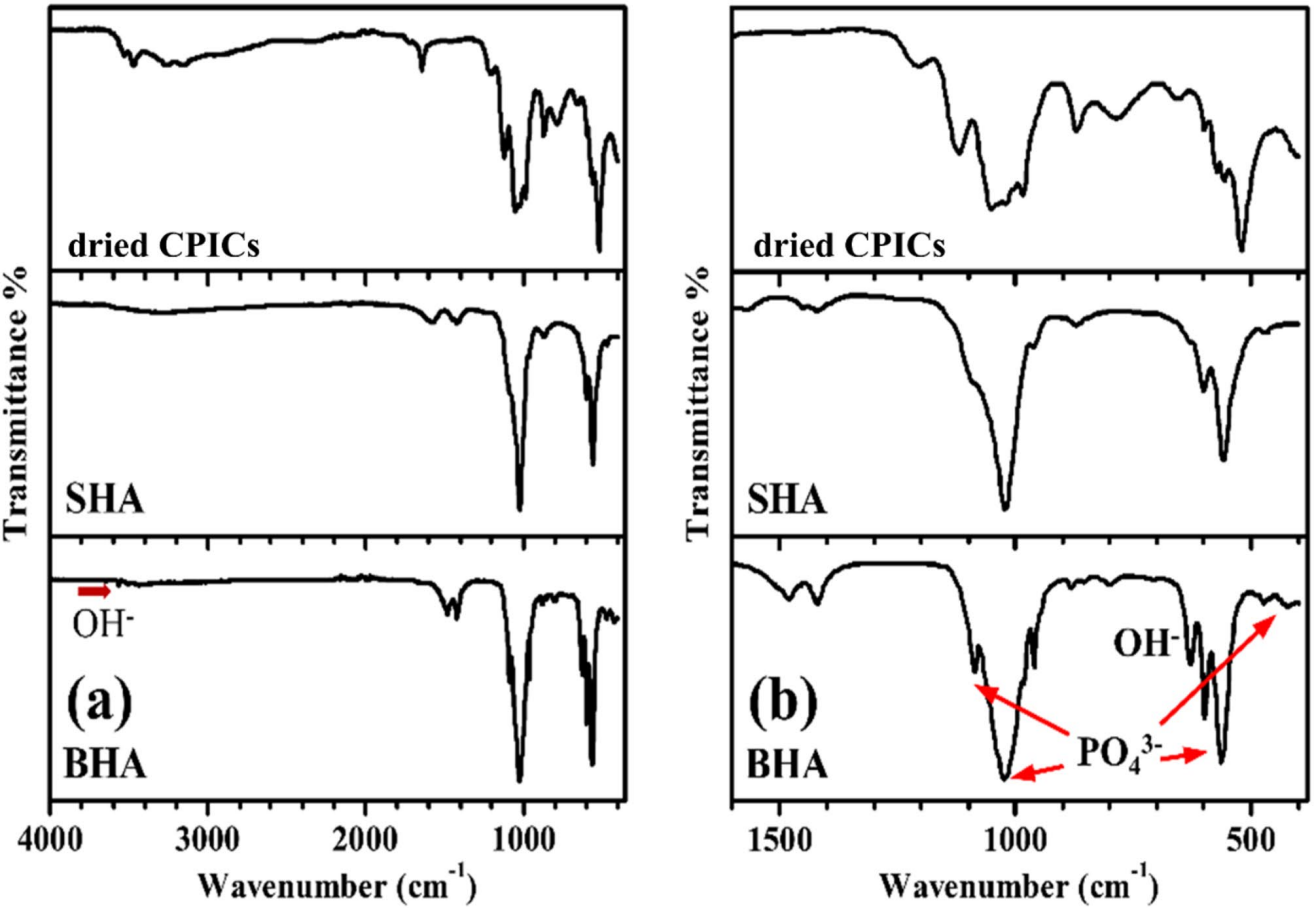
FTIR spectra of bone-derived hydroxyapatite (BHA), synthetic hydroxyapatite (SHA), and dried calcium phosphate ionic clusters (CPICs); (**a**) Full spectrum from 400 to 4000 cm^−1^ and (**b**) zooms in on the fingerprint regions.

The FTIR spectrum of BHA (Fig. 6a and b) shows all of the characteristic bands of enamel Hydroxyapatite (HAP) that includes OH^-^ peaks at 3565.6 and 628.8 cm^−1^, PO_4_^3−^ peaks at 1085.5, 1023.4, 960.3, 597.9, 562.4 and 472.6 cm^−1^^25,26^. The resonances at 1479.8 cm^−1^ were complex bands of type-A substitution (CO_3_^2−^ substitutes OH^−^) and type-B substitution (CO_3_^2−^ substitutes PO_4_^3−^)^27^. A small peak at 853.6 cm^−1^ is assigned to HPO_4_^2−^ indicating the presence of calcium-deficient HAP^28^.

#### Synthetic hydroxyapatite (SHA)

In the SEM image of SHA powder (Fig. 5), a bundle-like structure with lengths measuring 1 or 2 µm were observed. Each bundle is composed of multiple nanorods, which contribute to a relatively large surface area. This morphology was achieved by incorporating citric acid as a template during the formation of hydroxyapatite^22^. The specific morphology of SHA was chosen to increase the probability of entanglement between SHA and the newly formed calcium phosphate mass when combined with CPICs.

In the FTIR spectrum of SHA (Fig. 6a and b), it was found that the characteristic peaks of OH^-^ at 3572 and 634 cm^−1^ are absent. This absence might be due to the inclusion of the OH^-^ peak at 3572 cm^−1^ in the broad band of adsorbed water at 3240 cm^−1^, while the OH^-^ peak at 634 cm^−1^ might be overlapped by the PO_4_^3−^ band around 600 cm^−1^^29^. Peaks at 1021.5, 961.3, 600.6, 558.6, and 470.2 cm^−1^ are corresponding to PO_4_^3−^ and are considered characteristic of a typical hydroxyapatite (HAP) in the FTIR spectrum^26^. Peaks at 1569.1, 1449.1, and 1419.5 cm^−1^ were assigned to the CO_3_^2−^ group. The presence of carbonate ions may be due to the interaction between ambient carbon dioxide and the hydroxyapatite particles during processing^30,31^.

#### Dried calcium phosphate ionic clusters (CPICs)

In the SEM image of the dried CPICs, the powder manifests as agglomerated flakes, with each flake exceeding 1 µm in size (Fig. 5). As previously mentioned, dried CPICs were expected to be amorphous calcium phosphate (ACP)^8^. However, upon conducting FTIR examination of the dried CPICs (Fig. 6a and b), the characteristic rounded absorption bands of PO_4_^3−^ around 1000 cm^−1^ and 550 cm^−1^, which indicate the amorphous nature of ACP, were absent^32^. The OH^−^ peak was found around 3528.3 cm^−1^^29^, and the PO_4_^3−^ peaks were observed around 598 and 573 cm^−1^. Furthermore, specific absorption peaks associated with β-tricalcium phosphate (β-TCP) were present at 1118.3 and 983.7 cm^−1^^31,33^. A peak at 1202.6 cm^−1^, assigned to C–N, indicated the presence of the residual triethylamine (TEA) in the powder. The slight shift from the typical C–N peak, normally around 1200 cm^−1^, may be attributed to the interaction between the CPICs and TEA^8^. Based on the FTIR spectrum of the dried CPICs powder, it was concluded that the dried CPICs is not ACP, and it is suggested that the powder could be β-TCP or hydroxyapatite (HAP), or possibly a mixture of both. To confirm the nature and size of the dried CPICs powder, further investigations such as XRD and TEM were conducted.

**Fig. 7:**
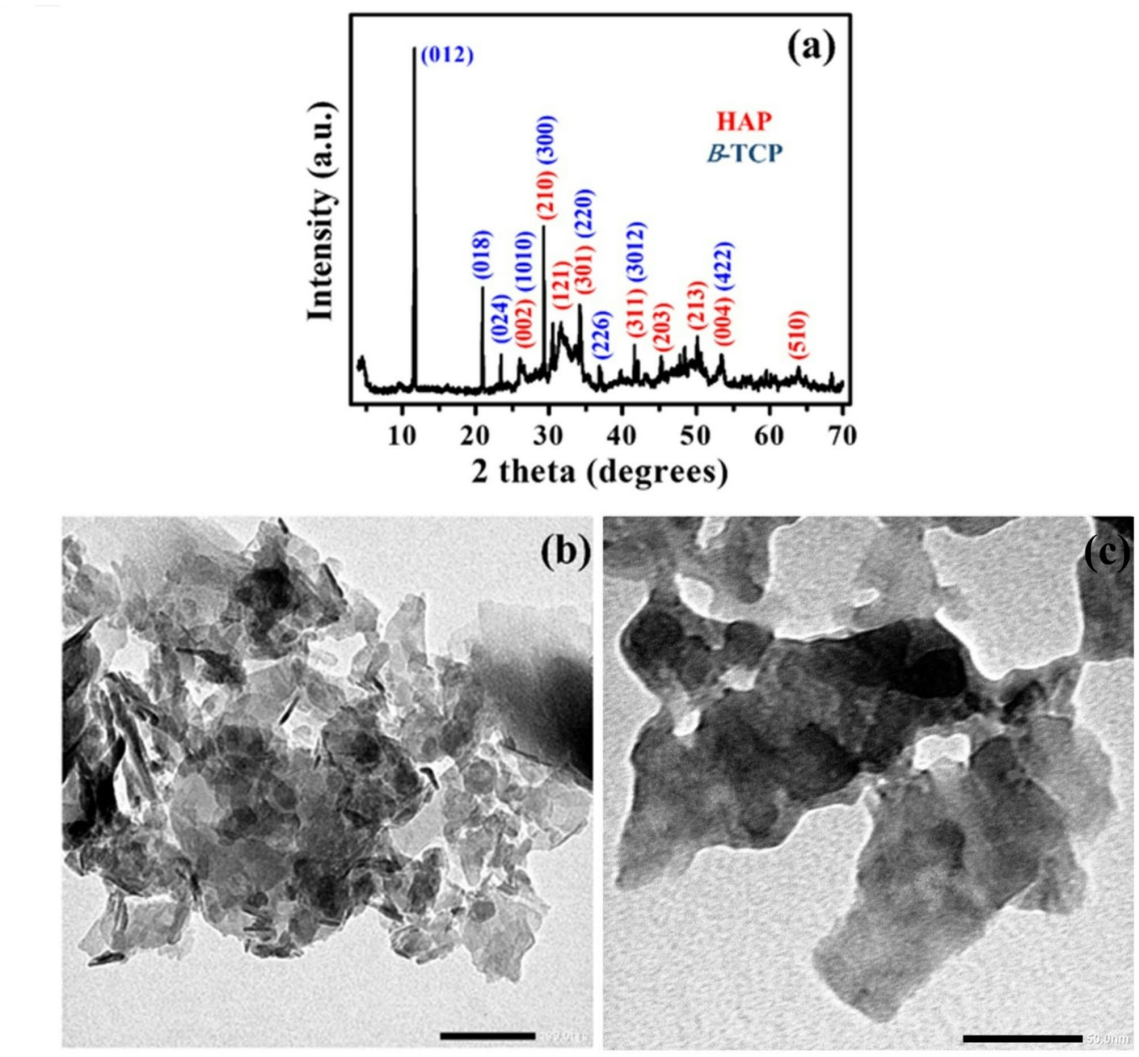
XRD pattern and TEM images of dried calcium phosphate ionic clusters (CPICs). XRD pattern of dried CPICs, Miller indices of the hydroxyapatite (HAP) crystal plane (red), Miller indices β-tricalcium phosphate (β-TCP) crystal planes (blue) (**a**). TEM images of dried CPICs; 100 nm scale bar (**b**) and 50 nm scale bar (**c**).

Figure 7a illustrates the XRD pattern of dried CPICs. By comparing it with the reference patterns of pure HAP and β-TCP that were obtained from the International Center for Diffraction Data (ICDD) database^34^, characteristic peaks corresponding to both phases (HAP and β-TCP) were identified. Notably, all the peaks that were observed in the dried CPICs XRD spectrum are sharp, indicating the absence of ACP^35^. The XRD pattern results indicate that the resulting powder is a mixture of β-TCP and HAP, which aligns with the findings of the FTIR analysis. This finding contradicts Shao et al.^8^, who reported that air drying of CPICs results in the formation of ACP.

The formation of a mixture of HAP and β-TCP, instead of ACP, can be attributed to the metastability of ACP. The metastability of ACP allows for its transformation into crystalline phases, including HAP or β-TCP, or both^36,37^. β-TCP can be considered a transitional phase in the transformation towards HAP^15^.

The TEM image of dried CPICs (Fig. 7b) indicates the presence of aggregated, flake-shaped, and nano-sheets with varying sizes. In Fig. 7c, the dried CPICs powder appears as polygonal nanosheets with well-defined boundaries. These sheets exhibit size variations ranging from 100 to 200 nm in length and 50 to 100 nm in width. The surface of the powder appears smooth without any noticeable irregularities or nano-scale texture. Notably, the observation of polygonal nanosheets, clear boundaries, and a smooth surface is consistent with previous studies on the TEM image of β-TCP^38,39^.

### Repaired enamel defects

In this study, the enamel defect was approximately 500 µm, so various calcium phosphate particles were combined with Calcium phosphate ionic clusters (CPICs) to create a material composed entirely of calcium phosphate to fill the defect. While CPICs have demonstrated an ability to promote structurally integrated and orderly mineral deposition on demineralized enamel surfaces^8,40,41^, the resulting regenerated layers remain relatively thin, limiting their application for the restoration of larger enamel defects. For example, Shao et al.^8^ reported a regenerated enamel layer of only 2.8 µm following a single application of TEA-stabilized CPICs on enamel etched with phosphoric acid for 30 s. Similarly, Wang et al.^40^ applied CPICs to enamel surfaces etched for 10 min and observed the formation of a continuous calcium phosphate layer with a thickness of less than 10 µm, which adhered well to the enamel substrate. Jiang et al.^41^ further confirmed the formation of a 2–3 µm thick layer of rod-like crystals in a fish-scale arrangement following CPICs application, highlighting the material’s capacity for biomimetic mineralization. Collectively, these studies show that while CPICs support nanoscale repair and surface integration, they do not provide sufficient volume to address larger, macroscale enamel cavities. Therefore, in this study, hydroxyapatite particles were incorporated alongside CPICs to enhance the overall mineral fill and enable a more comprehensive reconstruction of extensive enamel loss.

During biomineralization, the amorphous phase covers the crystalline mineral phase, leading to the epitaxial growth of the existing crystal^8^. In case of dental enamel, the amorphous phase is ACP and the crystalline phase is hydroxyapatite (HAP). However, the application of amorphous calcium phosphate (ACP) phase failed to generate the epitaxial growth of enamel HAP crystals. Even when using nanoscale ACP particles (~ 20 nm), these particles could be adsorbed and even assembled into HAP, but they did not cause the epitaxial growth of the crystals^42^. This may be due to coalescence and fusion between particles at the nanoscale^43^. As a result, researchers sought methods to establish an amorphous interface over the crystalline phase by utilizing the fundamental building blocks of ACP rather than ACP itself.

Calcium phosphate ionic clusters (CPICs), few nanometers in size, can serve as basic building blocks for ACP and HAP^44,45^. Based on the ionic inorganic polymerization strategy, a stabilizer was added to prevent these nanoclusters from spontaneous aggregation and nucleation^44^. Triethylamine (TEA) is a small organic molecule that is volatile in room temperature. It was successfully used to stabilize CPICs during enamel repair. Other irremovable organic additives cannot be used in enamel repair as it would interfere with the continuity of the enamel inorganic crystals^8^.

When the CPICs are introduced into the defect or mixed with the added calcium phosphate particles using either technique, they are exposed to air, causing the TEA and ethanol to evaporate. This evaporation process allows the calcium phosphate nanoclusters to aggregate and form ACP. The ACP coats the calcium phosphate crystals, potentially creating a uniform layer around them. This ACP coating may act as a precursor for the epitaxial growth of the existing crystals^8,46^. Inside the enamel defect, two types of crystals were coated with ACP: the enamel HAP in the floor and walls of the defect, and the added calcium phosphate particles (SHA, BHA, or dried CPICs). This mechanism allows the CPICs to induce epitaxial growth of the coated crystals and/or form an additional calcium phosphate phase between the existing particles, creating a cohesive new mass.

Two techniques were employed to mix the CPICs with the calcium phosphate particles: the LbL technique and the PM technique. The SEM images were employed to evaluate the adaptation between the newly formed calcium phosphate mass and the enamel to form structural integrity. Furthermore, FTIR analysis was performed on etched enamel as well as enamel treated with CPICs solution, CPICs with BHA, and CPICs mixed with dried CPICs to provide clearer evidence of structural changes following treatment.

#### Scanning electron microscopy (SEM) imaging

**Fig. 8:**
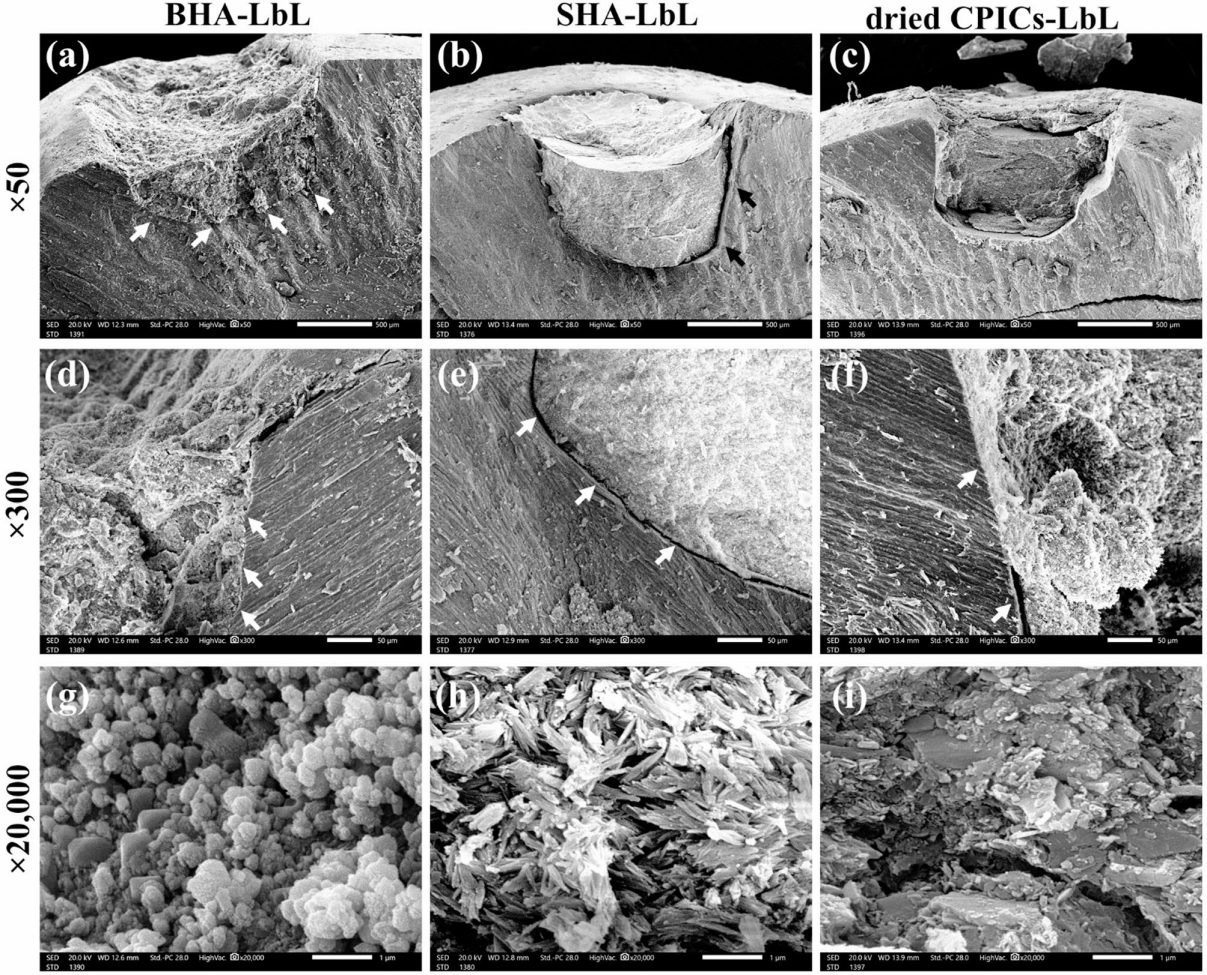
The SEM images of enamel defect repaired with bone-derived hydroxyapatite (BHA), synthetic hydroxyapatite (SHA), and dried calcium phosphate ionic clusters (CPICs) particles using LbL technique. Complete integration of BHA-LbL with enamel (**a**), integration failure between SHA-LbL and enamel (**b**), dried CPICs-LbL mass detachment (**c**), confirming complete integration of BHA-LbL (**d**), presence of 4 to 6 µm microleakage between SHA-LbL and enamel (**e**), integration failure and cohesion failure of dried CPICs-LbL (**f**), internal structure of BHA-LbL (**g**), internal structure of SHA-LbL (**h**), internal structure of dried CPICs-LbL (**i**). Scale bar; (**a**, **b**, and **c**; 500 µm), (**d**, **e**, and **f**; 50 µm) and (**g**, **h**, and **i**; 1 µm).

*Group I. LbL technique* Fig. 8 shows the repair of minor enamel defects using CPICs mixed with BHA, SHA, and dried CPICs through the LbL technique. In Fig. 8a at 50×magnification, the sample using BHA that was mixed with CPICs via the LbL technique (BHA-LbL) demonstrates excellent integration with the tooth structure, with no observable gaps. The higher magnification SEM image in Fig. 8d at 300 × confirms complete integration between BHA-LbL and enamel, although crystallographic continuity cannot be confirmed using SEM alone.

In contrast, there is a lack of integration between the mass formed by mixing CPICs with SHA via LbL technique (SHA-LbL) and the enamel. This lack of integration is evident from the presence of microleakage observed in Fig. 8b at 50×magnification. In the higher SEM image magnification (Fig. 8e at 300×), the width of the microleakage is measured to be approximately 4–6 µm. This observed microleakage suggests a potential shrinkage towards the mass center, pulling the mass away from the cavity wall. This shrinkage can be attributed to several factors. The most likely cause is the loss of volatile components, such as ethanol and TEA, during the reaction. Additionally, if there is a phase transformation, such as the transition from ACP to a more crystalline form, this could also contribute to shrinkage due to the volume reduction associated with crystallization. This shrinkage is more pronounced in SHA compared to BHA, due to the smaller size of SHA particles. Particles smaller in size have a higher surface area to volume ratio, which means that more surface is available for interactions with CPICs.

The difference in size and morphology between BHA and SHA also impacts the uniformity of the resulting mass. The BHA-LbL mass tends to have a rough surface, likely due to the larger BHA particles, some of which are at the microscale, protruding from the mass. In contrast, the SHA-LbL mass appears smooth and homogeneous. This smoothness is likely due to the smaller and more uniform morphology of SHA particles.

The dried CPICs-LbL sample exhibits poor adhesion and integration failure with the tooth structure. Figure 8c at 50×magnification shows detachment of the dried CPICs-LbL mass, indicating a lack of integration and adhesion. In Fig. 8f at 300×, cohesive failure inside the material is observed, as part of it remains attached and integrated with the tooth structure, while in other areas, detachment occurs due to a failure in adhesion between the dried-LbL mass and the tooth structure. These undesirable reactions could be due to the presence of various phases within the dried CPICs powder (HAP and β-TCP). Furthermore, the particle aggregation, observed in SEM and TEM images of dried CPICs, might obstruct the CPICs infiltration between particles. This could hinder the formation of a new calcium phosphate phase, potentially leading to cohesive and adhesive failure.

To examine the internal structure of BHA-LbL, SHA-LbL, and dried CPICs-LbL samples, SEM images at higher magnifications were analyzed. In the BHA-LbL sample, agglomerated globular particles of different sizes with some spaces between them are observed. Additionally, flake-like BHA particles are visible (Fig. 8g at 20,000**×**). Similarly, the SHA-LbL sample exhibits a bundle-like structure of SHA powders with multiple spaces between them (Fig. 8h at 20,000×). In the dried CPICs-LbL sample, more and larger spaces between the particles are observed inside the mass (Fig. 8i at 20,000×). The presence of flake-like BHA particles and the bundle structure of SHA particles indicate that the newly formed calcium phosphate phase, formed by CPICs after the evaporation of the TEA stabilizer, failed to completely cover the particles. Moreover, the presence of spaces between the particles suggests that the new calcium phosphate phase was unable to bind the particles and fill the gaps between them. The SEM images of enamel defects repaired with BHA, SHA, and dried CPICs powders using the LbL technique indicate incomplete coverage of the calcium phosphate particles by the CPICs. This results in incomplete binding of the particles together by the newly formed calcium phosphate phase. In terms of integration with the enamel surface, only BHA-LbL demonstrates maximum integration without any gaps.

*Group II: PM technique* To achieve optimal wetting of the particles with the CPICs ethanol solution, an alternative technique was proposed. This method involves mixing the powder with the CPICs ethanol solution to create a paste before application, thereby ensuring thorough wetting of the particles. Similar to the LbL technique, a drop of CPICs ethanol solution is first added, followed by the paste before the TEA evaporates. The amount of CPICs ethanol solution and the weight of the powder vary according to the powder density. Several trials were conducted to determine the optimal ratio for each type of particle to achieve a paste consistency that is easily manipulated and condensed into the defect. The paste was applied immediately after mixing to prevent TEA evaporation and ACP formation before application inside the defect. Based on the observed lack of adhesion and detachment of the dried CPICs-LbL sample, as mentioned earlier, it was determined that using dried CPICs particles with the PM technique was unsuitable. Therefore, only BHA and SHA powders were utilized with the PM technique.

**Fig. 9:**
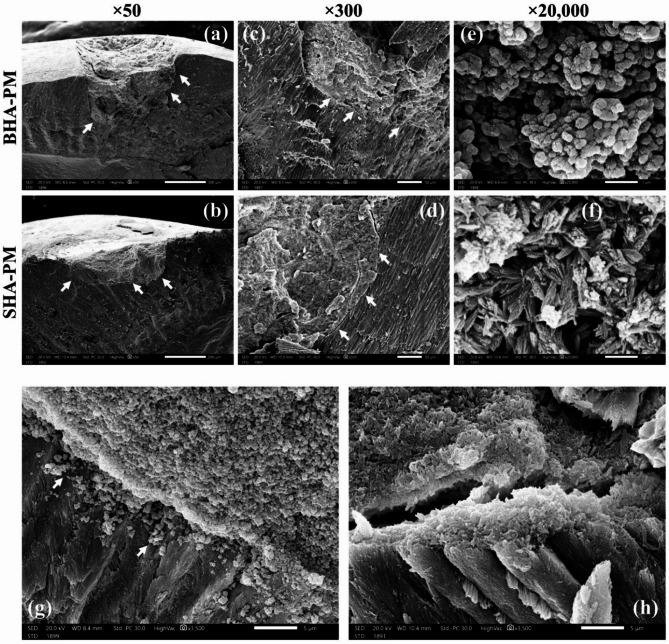
The SEM images of enamel defect repaired with bone-derived hydroxyapatite (BHA) and synthetic hydroxyapatite (SHA) using PM technique. Complete integration of BHA-PM with enamel; (**a**), smaller microleakage present between enamel and SHA-PM compared to SHA-LbL; (**b**), confirming complete integration of BHA-PM (**c**), microleakage measures less than 1 µm between SHA-PM and enamel; (**d**), internal structure of BHA-PM; (**e**), internal structure of SHA-PM; (**f**), interface between enamel and BHA-PM showing diffusion of calcium phosphate into underlying enamel; (**g**), interface between enamel and SHA-PM showing cohesive failure of SHA-PM with no diffused powders; (**h**). Scale bar; (**a** and **b**, 500 µm), (**c** and **d**, 50 µm), (**e** and **f**, 1 µm), and (**g** and **h**, 5 µm).

**Fig. 10:**
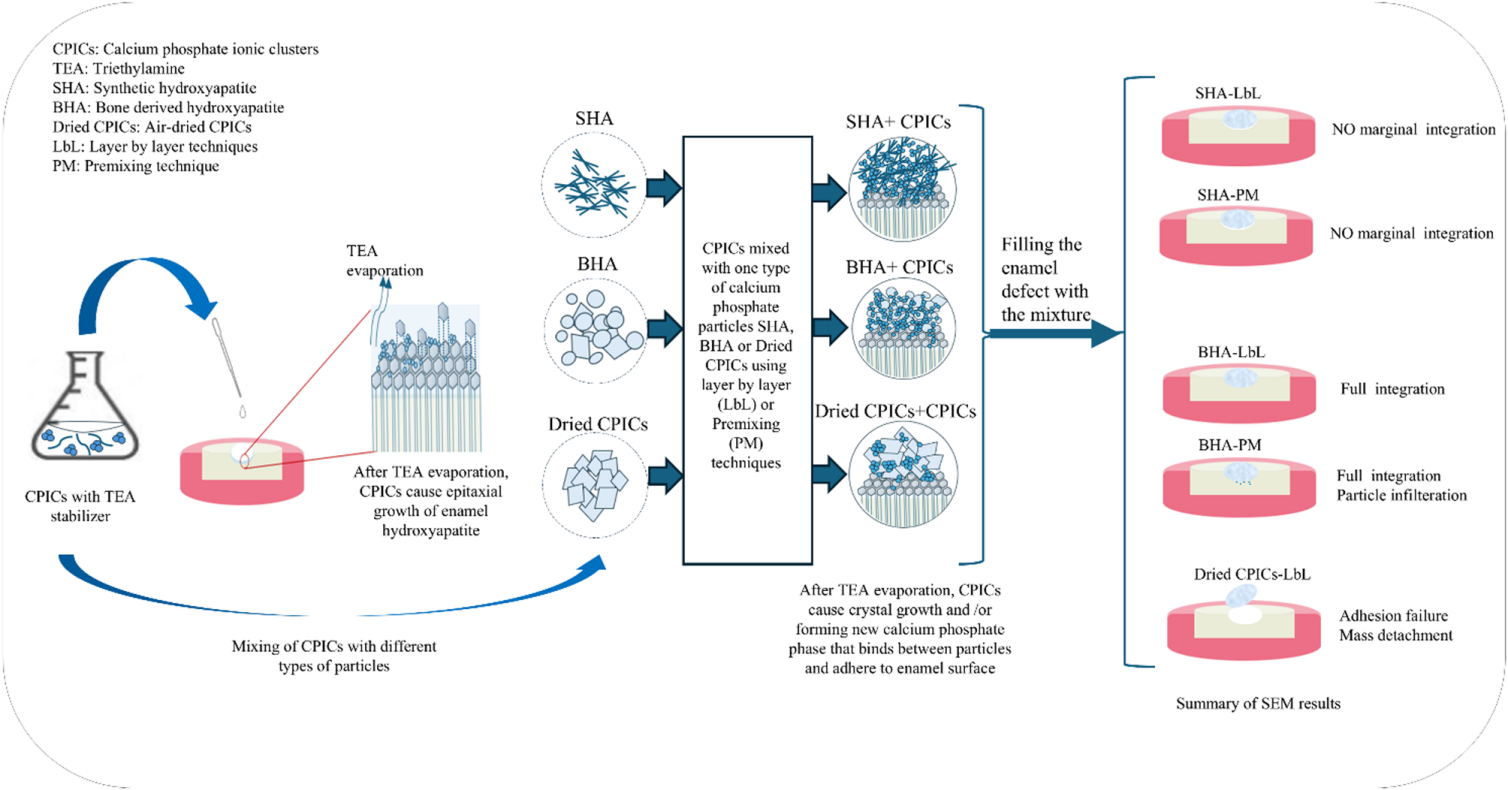
Schematic diagram shows the used mixing techniques and the corresponding morphology concluded from the SEM images.

Figure 9 shows the repair of minor enamel defects using CPICs mixed with BHA or SHA through the PM technique. Similar to BHA-LbL, the mass formed by mixing BHA with CPICs via PM technique (BHA-PM), demonstrated complete integration with the enamel, exhibiting no gaps (Fig. 9a at 50×magnification). The absence of microleakage between BHA-PM and enamel is confirmed by the higher magnification of SEM image in Fig. 9c at 300×. In comparison to SHA-LbL, smaller gap sizes are observed between the mass formed by mixing CPICs with SHA via PM technique (SHA-PM) and the enamel (Fig. 9b at 50×). In the higher magnification SEM image (Fig. 9d at 300×), the microleakage is measured to be less than 1 µm. The internal structure of BHA-PM and SHA-PM samples is examined using SEM images at greater magnification. Generally, the internal structure is denser compared to LbL technique. Unlike BHA-LbL, the BHA-PM sample does not exhibit a flake morphology of BHA powder (Fig. 9e at 20,000×), indicating complete coverage of the BHA particles by the new calcium phosphate phase. However, some spaces between powders are still visible. Comparatively, the SHA-PM sample shows more globular structures deposited on the SHA powders (Fig. 9f at 20,000×). SEM images at the interface between BHA-PM and the tooth structure reveal complete integration, with some particles appearing to penetrate into the enamel; however, diffusion into enamel cannot be confirmed without additional analytical methods (Fig. 9g at 3500×). When using SHA, particle diffusion is absent, and cohesive failure is evident by a fracture line within the mass, separating the newly integrated layer from the rest of the material (Fig. 9h at 3500×). SEM images of enamel defects repaired with BHA and SHA using the PM technique show a denser internal structure and better particle binding by the newly formed mass compared to the LbL technique. The microleakage between SHA-PM and enamel is also smaller than that seen with SHA-LbL. BHA-PM exhibits complete integration with the enamel, along with the diffusion of calcium phosphate particles into the underlying enamel. The differences between the two techniques may be due to the increased amount of CPICs infiltrated between the nanoparticles, facilitating the formation of additional calcium phosphate material, such as a new layer of hydroxyapatite or other related phases. Figure 10 summarizes the mixing techniques and the corresponding SEM results.

Upon visual inspection, all the newly formed calcium phosphate masses appeared opaque and white. Additionally, they could be easily scratched with manual probing, indicating weak mechanical properties.

The weak mechanical properties of the newly formed mass suggest that crystallization have occurred via the classical nucleation pathway.

The early stage of crystallization is always referred to as nucleation^47^. Nucleation pathways describe the various mechanisms through which new crystals or phases begin to form. They are generally divided into classical and nonclassical pathways, each with distinct features that influence the overall crystallization behavior^48^. Classical crystallization involves atoms, ions, or molecules systematically and orderly coming together to form a crystal lattice. Unlike classical models, nonclassical nucleation mechanisms often follow more complex routes, typically involving intermediate metastable phases. These mechanisms support multistep transitions instead of the direct formation of crystals seen in classical pathways^49^. In classical nucleation theory, nucleation process is governed by fluctuations within the supersaturated solution, leading to the formation of small crystalline nuclei. Because of the inherent limitations of classical crystallization, materials that were produced through this pathway often result in powder particles rather than bulk solid form^50,51^. Powdery crystal formation often originates during the nucleation stage, especially when rapid nucleation produces many small crystals instead of fewer, more stable ones. Without precise control, this can yield loosely bound, fine particles that lack cohesion and result in a powder-like appearance^48,52^.

Although the inorganic ionic polymerization approach used in this study should overcome the limitation of the classical crystallization pathway^9,53^ and allow the existence of intermediate phase as proposed by the non-classical crystallization theory^9^, the study’s findings defy this prediction. The conditions that favor non-classical nucleation are multifaceted and include factors such as supersaturation and temperature^49,54^. Temperature plays a crucial role in determining which nucleation mechanism predominates; lower temperatures are generally more conducive to non-classical pathways, while higher temperatures favor classical mechanisms^55^. Till now, the exact nucleation pathway and detailed kinetics remain unclear, and the coexistence of different nucleation pathways is a possibility^56^.

#### FTIR analysis

**Fig. 11:**
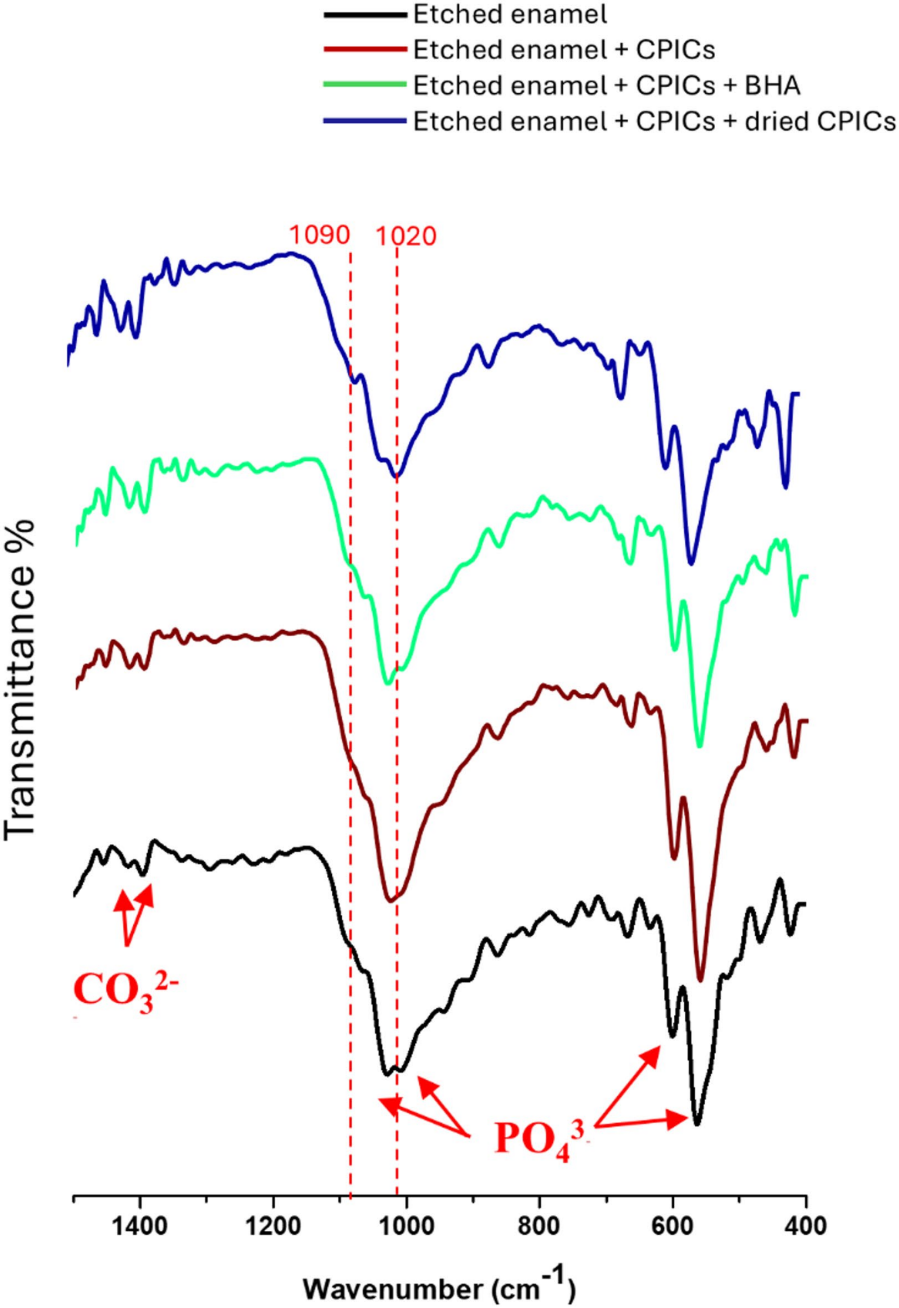
FTIR spectra of etched enamel (black), etched enamel treated with CPICs solution (red), etched enamel treated with CPICs mixed with BHA (green) and etched enamel treated with CPICs mixed with dried CPICs powder (blue).

Figure 11 displays the FTIR spectra (400–1500 cm^−^1) of etched enamel surface before and after the application of CPICs solution alone, CPICs mixed with BHA, CPICs mixed with dried CPICs powder. All spectra are characterized by prominent absorption bands corresponding to phosphate and carbonate groups, which are indicative of the hydroxyapatite structure. The peak at 1020 cm^−1^ is absent in the spectrum of etched enamel treated with CPICs mixed with BHA, indicating enhanced crystallization and an increased mineral content. This peak is typically associated with phosphate vibrations in poorly crystalline, non-stoichiometric apatites, whereas the peak at 1030 cm^−^1 corresponds to more crystalline, stoichiometric apatites^57^. The absence of the 1020 cm^−^1 peak thus reflects a shift toward a more ordered and stoichiometric hydroxyapatite structure. Furthermore, bands near 1100 cm^−1^ were observed exclusively in etched enamel treated with CPICs mixed with dried CPICs powder. These bands are characteristic of non-stoichiometric apatites containing HPO₄2− ions and are absent in well-crystallized, stoichiometric hydroxyapatite^58^. No other significant spectral differences were detected between the etched enamel and the treated samples.

The limitations of this study include the exclusive use of SEM for evaluating the material’s performance. While SEM provided valuable insights into the structural integration and marginal adaptation of the material, it does not offer comprehensive information on remineralization efficiency or chemical composition. Incorporating advanced analytical techniques such as micro-computed tomography (micro-CT) and Raman spectroscopy could enhance the evaluation by providing detailed data on material composition and its remineralization potential.

Future research should also focus on assessing the solubility of the material in saliva and its behavior under physiological conditions, including pH cycling that simulates the oral environment, over an extended period of time. Additionally, innovative approaches should be explored to enhance the mechanical properties of the material, ensuring greater durability and clinical applicability.

## Conclusion and clinical relevance

Based on the SEM images of all the studied groups; using BHA leads to complete integration with the underlying enamel in both techniques (LbL and PM), without any evidence of cohesive failure. Furthermore, the group utilizing BHA with the PM technique is the only method that demonstrates particle infiltration into the underlying enamel. In addition, based on the FTIR analysis, the application of BHA-PM appears to enhance the crystallinity of the underlying enamel and increases its mineral content. However, the extent of remineralization requires further confirmation through additional quantitative analyses, which were not included in the present study. If future studies verify that a significant level of remineralization occurs, BHA-PM may offer the dual benefit of promoting enamel repair while achieving integration with the native tooth structure. Consequently, it could serve as a temporary or intermediate material for the treatment of demineralized enamel prior to the placement of a definitive resin restoration. This approach is particularly beneficial as the bond strength of adhesive materials increases with the presence of minerals in the tooth structure^59,60^. However, the newly formed calcium phosphate mass displayed low mechanical strength as it could be easily scratched with manual probing. These observations suggest that the new calcium phosphate phase, formed after the evaporation of TEA, inadequately bounds the calcium phosphate particles together and failed to generate a cohesive and robust mass. Gaining a deeper understanding of the crystallization mechanisms and conducting further investigations could lead to improvements in the mechanical properties of the resulting material, potentially allowing its use as a permanent restoration.

## Data Availability

All data generated or analyzed during this study are included in this published article.

## References

[1] Elsharkawy, S. & Mata, A. Hierarchical biomineralization: From nature’s designs to synthetic materials for regenerative medicine and dentistry. *Adv. Healthc. Mater.* **7**(18), 1800178. 10.1002/adhm.201800178 (2018). 10.1002/adhm.201800178 29943412

[2] Tang, Z. H., Shan, S. Z., Chen, Z. & Shao, C. Y. Progress in the application of biomimetic mineralization for tooth repair. *Minerals* **13**(11), 1433. 10.3390/min13111433 (2023).

[3] Liu, L., Shi, Z. & Wu, J. Advances in biomimetic mineralization of tooth enamel based on cell-free strategies. In *MATEC Web of Conferences* vol. 363 01032 (2022). 10.1051/matecconf/202236301032

[4] Yao, S. S. et al. Biomineralization: From material tactics to biological strategy. *Adv. Mater.* **29**(14), 1605903. 10.1002/adma.201605903 (2017). 10.1002/adma.201605903 28229486

[5] Zhang, J., Fang, W., Liu, Z. & Tang, R. Inorganic ionic polymerization: A bioinspired strategy for material preparation. *Biogeotechnics* **1**(1), 100004. 10.1016/j.bgtech.2023.100004 (2023).

[6] Gower, L. B. & Odom, D. J. Deposition of calcium carbonate films by a polymer-induced liquid-precursor (PILP) process. *J. Cryst. Growth* **210**(4), 719–734. 10.1016/s0022-0248(99)00749-6 (2000).

[7] Tang, S., Dong, Z., Ke, X., Luo, J. & Li, J. Advances in biomineralization-inspired materials for hard tissue repair. *Int. J. Oral Sci.* **13**(1), 42. 10.1038/s41368-021-00147-z (2021). 34876550 10.1038/s41368-021-00147-z PMC8651686

[8] Shao, C. Y. et al. Repair of tooth enamel by a biomimetic mineralization frontier ensuring epitaxial growth. *Sci. Adv.* **5**(8), eaaw9569. 10.1126/sciadv.aaw9569 (2019). 31497647 10.1126/sciadv.aaw9569 PMC6716959

[9] Wang, Q., Hu, L. S., Wang, X. Y. & Tang, R. K. Expanding from materials to biology inspired by biomineralization. *Interdiscip. Mater.* **3**(2), 165–188. 10.1002/idm2.12144 (2024).

[10] Pepla, E., Besharat, L. K., Palaia, G., Tenore, G. & Migliau, G. Nano-hydroxyapatite and its applications in preventive, restorative and regenerative dentistry: A review of literature. *Ann. Stomatol.* **5**(3), 108–114 (2014) (**in eng**). PMC4252862 25506416

[11] Sadat-Shojai, M., Khorasani, M. T., Dinpanah-Khoshdargi, E. & Jamshidi, A. Synthesis methods for nanosized hydroxyapatite with diverse structures. *Acta Biomater.* **9**(8), 7591–7621. 10.1016/j.actbio.2013.04.012 (2013). 23583646 10.1016/j.actbio.2013.04.012

[12] Wan, L. L., Cui, B. F. & Wang, L. J. A review on preparation raw materials, synthesis methods, and modifications of hydroxyapatite as well as their environmental applications. *Sustain. Chem. Pharm.* **38**, 101447. 10.1016/j.scp.2024.101447 (2024).

[13] Pu’ad, N., Koshy, P., Abdullah, H. Z., Idris, M. I. & Lee, T. C. Syntheses of hydroxyapatite from natural sources. *Heliyon* **5**(5), e01588. 10.1016/j.heliyon.2019.e01588 (2019). 31080905 10.1016/j.heliyon.2019.e01588 PMC6507053

[14] Ramesh, N., Ratnayake, J. T. B., Moratti, S. C. & Dias, G. J. Effect of chitosan infiltration on hydroxyapatite scaffolds derived from New Zealand bovine cancellous bones for bone regeneration. *Int. J. Biol. Macromol.* **160**, 1009–1020. 10.1016/j.ijbiomac.2020.05.269 (2020). 32504711 10.1016/j.ijbiomac.2020.05.269

[15] Ratnayake, J. T. B., Mucalo, M. & Dias, G. J. Substituted hydroxyapatites for bone regeneration: A review of current trends. *J. Biomed. Mater. Res. Part B-Appl. Biomater.* **105**(5), 1285–1299. 10.1002/jbm.b.33651 (2017). 10.1002/jbm.b.33651 26991026

[16] Liang, K. N. et al. Dental remineralization via poly(amido amine) and restorative materials containing calcium phosphate nanoparticles. *Int. J. Oral Sci.* **11**, 15. 10.1038/s41368-019-0048-z (2019). 31068570 10.1038/s41368-019-0048-z PMC6506538

[17] Moreau, J. L., Sun, L. M., Chow, L. C. & Xu, H. H. K. Mechanical and acid neutralizing properties and bacteria inhibition of amorphous calcium phosphate dental nanocomposite. *J. Biomed. Mater. Res. Part B-Appl. Biomater.* **98B**(1), 80–88. 10.1002/jbm.b.31834 (2011). 10.1002/jbm.b.31834 PMC3375606 21504057

[18] Devadiga, D., Shetty, P., Hegde, M. N. & Reddy, U. Bioactive remineralization of dentin surface with calcium phosphate-based agents: An in vitro analysis. *J. Conserv. Dent. JCD* **25**(1), 93–97. 10.4103/jcd.jcd_583_21 (2022) (**in eng**). 35722070 10.4103/jcd.jcd_583_21 PMC9200173

[19] Bohner, M., Santoni, B. L. G. & Döbelin, N. β-tricalcium phosphate for bone substitution: Synthesis and properties. *Acta Biomater.* **113**, 23–41. 10.1016/j.actbio.2020.06.022 (2020). 32565369 10.1016/j.actbio.2020.06.022

[20] Sandhu, S. V. et al. Sterilization of extracted human teeth: A comparative analysis. *J. Oral Biol. Craniofac. Res.* **2**(3), 170–175. 10.1016/j.jobcr.2012.09.002 (2012) (**in eng**). 25737861 10.1016/j.jobcr.2012.09.002 PMC3942122

[21] Kharouf, N. et al. Effectiveness of etching with phosphoric acid when associated with rubbing technique. *J. Stomatol.* **74**(1), 16–21. 10.5114/jos.2021.104693 (2021).

[22] Snihirova, D. et al. Hydroxyapatite microparticles as feedback-active reservoirs of corrosion inhibitors. *ACS Appl. Mater. Interfaces* **2**(11), 3011–3022. 10.1021/am1005942 (2010) (**in eng**). 20942404 10.1021/am1005942

[23] Barakat, N. A. M., Khil, M. S., Omran, A. M., Sheikh, F. A. & Kim, H. Y. Extraction of pure natural hydroxyapatite from the bovine bones bio waste by three different methods. *J. Mater. Process. Technol.* **209**(7), 3408–3415. 10.1016/j.jmatprotec.2008.07.040 (2009).

[24] I. ISO, "TS 80004-1: Nanotechnologies—Vocabulary—Part 1: Core Terms," *ISO (the International Organization for Standardization)* (2015).

[25] Chen, F. F. et al. "Low-cost and scaled-up production of fluorine-free, substrate-independent, large-area superhydrophobic coatings based on hydroxyapatite nanowire bundles. *Chemistry* **24**(2), 416–424. 10.1002/chem.201703894 (2018) (**in eng**). 29072343 10.1002/chem.201703894

[26] Ye, F., Guo, H., Zhang, H. & He, X. Polymeric micelle-templated synthesis of hydroxyapatite hollow nanoparticles for a drug delivery system. *Acta Biomater.* **6**(6), 2212–2218. 10.1016/j.actbio.2009.12.014 (2010) (**in eng**). 20004747 10.1016/j.actbio.2009.12.014

[27] Yang, W. H., Xi, X. F., Li, J. F. & Cai, K. Y. Comparison of crystal structure between carbonated hydroxyapatite and natural bone apatite with theoretical calculation. *Asian J. Chem.* **25**(7), 3673–3678. 10.14233/ajchem.2013.13709 (2013).

[28] Wen, Z. et al. Manipulation of partially oriented hydroxyapatite building blocks to form flowerlike bundles without acid-base regulation. *Colloids Surf. B Biointerfaces* **142**, 74–80. 10.1016/j.colsurfb.2016.02.016 (2016). 26930036 10.1016/j.colsurfb.2016.02.016

[29] Gheisari, H., Karamian, E. & Abdellahi, M. A novel hydroxyapatite—Hardystonite nanocomposite ceramic. *Ceram. Int.* **41**(4), 5967–5975. 10.1016/j.ceramint.2015.01.033 (2015).

[30] Anwar, A., Akbar, S., Sadiqa, A. & Kazmi, M. Novel continuous flow synthesis, characterization and antibacterial studies of nanoscale zinc substituted hydroxyapatite bioceramics. *Inorg. Chim. Acta* **453**, 16–22. 10.1016/j.ica.2016.07.041 (2016).

[31] Wang, A. et al. Effects of organic modifiers on the size-controlled synthesis of hydroxyapatite nanorods. *Appl. Surf. Sci.* **253**(6), 3311–3316. 10.1016/j.apsusc.2006.07.025 (2007).

[32] Vecstaudza, J., Gasik, M. & Locs, J. Amorphous calcium phosphate materials: Formation, structure and thermal behaviour. *J. Eur. Ceram. Soc.* **39**(4), 1642–1649. 10.1016/j.jeurceramsoc.2018.11.003 (2019).

[33] Hassan, M. N., Mahmoud, M. M., El-Fattah, A. A. & Kandil, S. Microwave rapid conversion of sol–gel-derived hydroxyapatite into β-tricalcium phosphate. *J. Sol-Gel. Sci. Technol.* **76**(1), 74–81. 10.1007/s10971-015-3753-x (2015).

[34] Ebrahimi, M. & Botelho, M. Biphasic calcium phosphates (BCP) of hydroxyapatite (HA) and tricalcium phosphate (TCP) as bone substitutes: Importance of physicochemical characterizations in biomaterials studies. *Data Brief* **10**, 93–97. 10.1016/j.dib.2016.11.080 (2017) (**in eng**). 27981198 10.1016/j.dib.2016.11.080 PMC5144648

[35] Eanes, E. D. Amorphous calcium phosphate: Thermodynamic and kinetic considerations. In *Calcium Phosphates in Biological and Industrial Systems* (ed. Amjad, Z.) 21–39 (Springer, 1998).

[36] Kim, S., Ryu, H.-S., Shin, H., Jung, H. S. & Hong, K. S. In situ observation of hydroxyapatite nanocrystal formation from amorphous calcium phosphate in calcium-rich solutions. *Mater. Chem. Phys.* **91**(2), 500–506. 10.1016/j.matchemphys.2004.12.016 (2005).

[37] Liu, S. et al. Effect of PEG amount in amorphous calcium phosphate on its crystallized products. *J. Mater. Sci. Mater. Med.* **20**(1), 359–363. 10.1007/s10856-008-3584-1 (2009). 18807264 10.1007/s10856-008-3584-1

[38] Fengcang, M. & Ping, L. Surface modification of β-TCP with stearic acid and its effects on β-TCP/PLLA biodegradable composite nanofibers. *J. Bone Rep. Recomm.* **02**(01), 2016. 10.4172/2469-6684.100020 (2016).

[39] Haghshenas, M. Mechanical characteristics of biodegradable magnesium matrix composites: A review. *J. Magnes. Alloys* **5**(2), 189–201. 10.1016/j.jma.2017.05.001 (2017).

[40] Wang, C. H., Mutalik, C., Yougbaré, S., Teng, N. C. & Kuo, T. R. Calcium phosphate nanoclusters for the repair of tooth enamel erosion. *Nanomaterials*10.3390/nano12121997 (2022). 35745336 10.3390/nano12121997 PMC9230511

[41] Jiang, W. et al. The effect of calcium phosphate ion clusters in enhancing enamel conditions versus Duraphat and Icon. *Aust. Endod. J.* **49**(S1), 46–57. 10.1111/aej.12689 (2023). 36127810 10.1111/aej.12689

[42] Li, L. et al. Repair of enamel by using hydroxyapatite nanoparticles as the building blocks. *J. Mater. Chem.* **18**(34), 4079–4084. 10.1039/B806090H (2008).

[43] Perez, A. et al. Cluster assembled materials: A novel class of nanostructured solids with original structures and properties. *J. Phys. D Appl. Phys.* **30**(5), 709. 10.1088/0022-3727/30/5/003 (1997).

[44] Habraken, W. J. E. M. et al. Ion-association complexes unite classical and non-classical theories for the biomimetic nucleation of calcium phosphate. *Nat. Commun.* **4**(1), 1507. 10.1038/ncomms2490 (2013). 23422675 10.1038/ncomms2490

[45] Onuma, K. & Ito, A. Cluster growth model for hydroxyapatite. *Chem. Mater.* **10**(11), 3346–3351. 10.1021/cm980062c (1998).

[46] DeVol, R. T. et al. Nanoscale transforming mineral phases in fresh nacre. *J. Am. Chem. Soc.* **137**(41), 13325–13333. 10.1021/jacs.5b07931 (2015). 26403582 10.1021/jacs.5b07931

[47] Lee, J., Yang, J., Kwon, S. G. & Hyeon, T. Nonclassical nucleation and growth of inorganic nanoparticles. *Nat. Rev. Mater.* **1**(8), 16034. 10.1038/natrevmats.2016.34 (2016).

[48] Jun, Y.-S. et al. Classical and Nonclassical Nucleation and Growth Mechanisms for Nanoparticle Formation. *Annu. Rev. Phys. Chem.* **73**, 453–477. 10.1146/annurev-physchem-082720-100947 (2022). 35113740 10.1146/annurev-physchem-082720-100947

[49] Smeets, P. J. M. et al. A classical view on nonclassical nucleation. *Proc. Natl. Acad. Sci.* **114**(38), E7882–E7890. 10.1073/pnas.1700342114 (2017). 28874584 10.1073/pnas.1700342114 PMC5617248

[50] Olafson, K. N., Li, R., Alamani, B. G. & Rimer, J. D. Engineering crystal modifiers: Bridging classical and nonclassical crystallization. *Chem. Mater.* **28**(23), 8453–8465. 10.1021/acs.chemmater.6b03550 (2016).

[51] Lewis, J. A. Colloidal processing of ceramics. *J. Am. Ceram. Soc.* **83**(10), 2341–2359. 10.1111/j.1151-2916.2000.tb01560.x (2000).

[52] Fan, X. et al. Effect of crystallization temperature, stirring rate, and pH on the crystallization of ammonium sulfate in the presence of calcium sulfate. *Phosphorus Sulfur Silicon Relat. Elem.* **200**(4), 383–395. 10.1080/10426507.2025.2482879 (2025).

[53] Giannoudis, P. V., Dinopoulos, H. & Tsiridis, E. Bone substitutes: An update. *Injury* **36**(3 Supplement), S20–S27. 10.1016/j.injury.2005.07.029 (2005). 16188545 10.1016/j.injury.2005.07.029

[54] Fu, H., Guan, B., Jiang, G., Yates, M. Z. & Wu, Z. Effect of supersaturation on competitive nucleation of CaSO_4_ phases in a concentrated CaCl_2_ solution. *Cryst. Growth Des.* **12**(3), 1388–1394. 10.1021/cg201493w (2012).

[55] You, H. & Fang, J. Particle-mediated nucleation and growth of solution-synthesized metal nanocrystals: A new story beyond the LaMer curve. *Nano Today* **11**(2), 145–167. 10.1016/j.nantod.2016.04.003 (2016).

[56] Jin, B., Liu, Z. M. & Tang, R. K. Recent experimental explorations of non-classical nucleation. *CrystEngComm* **22**(24), 4057–4073. 10.1039/d0ce00480d (2020).

[57] Carden, A. & Morris, M. D. Application of vibrational spectroscopy to the study of mineralized tissues. *J. Biomed. Opt.* **5**(3), 259–268. 10.1117/1.429994 (2000) (**in eng**). 10958610 10.1117/1.429994

[58] Rey, C., Shimizu, M., Collins, B. & Glimcher, M. J. Resolution-enhanced Fourier transform infrared spectroscopy study of the environment of phosphate ion in the early deposits of a solid phase of calcium phosphate in bone and enamel and their evolution with age: 2. Investigations in the nu3PO4 domain. *Calcif. Tissue Int.* **49**(6), 383–388. 10.1007/bf02555847 (1991). 1818762 10.1007/BF02555847

[59] Zafar, M. S. & Ahmed, N. The effects of acid etching time on surface mechanical properties of dental hard tissues. *Dent. Mater. J.* **34**(3), 315–320. 10.4012/dmj.2014-083 (2015) (**in eng**). 25904167 10.4012/dmj.2014-083

[60] Enan, E., Tawfik, M. A., Mehesen, R. & Basha, S. Remineralization potential and shear bond strength of surface treated hypomineralized enamel in bonding of orthodontic brackets: An in vitro study. *J. Adv. Oral Res.* **12**(1), 127–133. 10.1177/2320206820977734 (2021).

